# A Delphi Study to Identify Priority Indicators for Formulary Selection of Innovative Anticancer Drugs in China

**DOI:** 10.1111/hex.70652

**Published:** 2026-04-11

**Authors:** Jie Zhao, Youhong Hu, Yumei Zhu, Bin Wu, Ming Hu, Min Li, Yan Liang, Jia Li, Gaoxing Qiao, Xintong Fan, Helei Yan, Xiaoyun Wang, Zhixin Zheng, Xiaodan Zhang, Junwei Li, Tingting Lou, Yuchen Ma

**Affiliations:** ^1^ Department of Pharmacy The First Affiliated Hospital of Zhengzhou University Zhengzhou China; ^2^ School of International Pharmaceutical Business China Pharmaceutical University Nanjing China; ^3^ Department of Pharmacy, Shanghai Chest Hospital Shanghai Jiao Tong University School of Medicine Shanghai China; ^4^ West China School of Pharmacy Sichuan University Chengdu China; ^5^ Department of Pharmacy Nanyang Central Hospital Nanyang China; ^6^ Department of Pharmacy Anhui Provincial Cancer Hospital Hefei China; ^7^ Department of Pharmacy The Fifth Affiliated Hospital of Zhengzhou University Zhengzhou China; ^8^ Department of Pharmacy Huaihe Hospital of Henan University Kaifeng China; ^9^ Department of Pharmacy Xuchang Central Hospital Xuchang China

**Keywords:** decision framework, Delphi methodology, hospital formulary selection, innovative anticancer drug, value assessment

## Abstract

**Objective:**

To develop a multi‐stakeholder, consensus‐based decision support framework for prioritizing innovative anticancer drugs in Chinese hospital formularies, balancing clinical benefit, patient access, and financial sustainability.

**Methods:**

A two‐round Delphi survey was conducted with a multidisciplinary expert panel (*n* = 62 in Round 1; *n* = 51 in Round 2, 82.3% retention rate) comprising clinicians, pharmacists, health administrators, and patient representatives. The initial set of 19 indicators across 9 dimensions, derived from a literature review, was iteratively refined based on panel feedback. Consensus was defined as ≥ 80% of respondents rating an indicator as 4 or 5 on a 5‐point Likert scale, dispersion was summarized by interquartile range (IQR).

**Results:**

The panel reached a clear consensus on a structured framework comprising seven key dimensions and 16 measurable indicators. The top three prioritized dimensions were clinical benefits, safety, and economic impact. The framework incorporates policy‐sensitive indicators relevant to China's healthcare system, such as pharmaceutical access and access barriers of genetic testing. Five indicators from the initial list were discarded, and two new ones were added based on expert input, with specific metrics refined for enhanced objectivity and practicality (e.g., “patient out‐of‐pocket expenditure per year”).

**Conclusion:**

This study presents a transparent, standardized, and actionable decision‐making framework to support formulary selection of innovative anticancer drugs in Chinese hospitals—particularly in resource‐adequate settings. By incorporating diverse stakeholder perspectives and establishing clear evaluation criteria, the framework reduces subjectivity and supports consistent value assessment. It provides practical guidance for hospital pharmacy and therapeutics committees and contributes to more equitable, evidence‐informed, and financially sustainable drug policy development in China.

**Patient or Public Contribution:**

Patient representatives participated in the first‐round expert panel, where they rated candidate indicators, shared their lived experiences, and provided feedback on the interview guides and questionnaires.

**Abbreviations**
AHP: analytic hierarchy process
ASCO: American Society of Clinical Oncology
BIA: budget impact analysis
CEA: cost‐effectiveness analysis
CNKI: China National Knowledge Infrastructure
CUA: cost‐utility analysis
DRG: diagnosis‐related group
EMR: electronic medical record
ESMO: European Society for Medical Oncology
HIS: hospital information system
HTA: Health Technology Assessment
IQR: interquartile range
ISPOR: The International Society for Pharmacoeconomics and Outcomes Research
MCDA: Multiple Criteria Decision Analysis
NCCN: National Comprehensive Cancer Network
NRDL: National Reimbursement Drug List
PRO: patient‐reported outcomes
P&T: pharmacy & therapeutics
VAF: value assessment framework

## 1 Introduction

The rapid emergence of innovative anticancer agents—defined as novel therapeutic agents with distinct mechanisms of action, including targeted therapies, immune checkpoint inhibitors and cell therapies applicable to antitumor treatment—has profoundly improved outcomes for cancer patients [1, 2, 3]. However, their high costs and the dynamic nature of their clinical evidence (e.g., phased approvals and immature comparative data) pose significant challenges for healthcare systems striving to balance innovation, affordability, and sustainability [4, 5, 6, 7].

Hospital formularies serve as a core instrument for drug selection and resource management. In practice, however, formulary decisions are often constrained by budget silos, such as diagnosis‐related group (DRG) payment caps, leading to an overemphasis on drug “price” at the expense of a comprehensive assessment of multidimensional “value” [8, 9]. This underscores the urgent need to transform the formulary from a mechanism of “passive cost control” into a framework for “active optimization of health outcomes.”

Value assessment frameworks (VAFs) have been developed internationally to support such decisions, including those by ISPOR, NCCN, ASCO, and ESMO, which inform pricing, reimbursement, and procurement [10, 11, 12, 13, 14, 15, 16, 17]. Specific frameworks for formulary selection have also been proposed in contexts such as Canada and Indonesia [18, 19]. While instructive, these existing VAFs often exhibit critical limitations when considered for direct application in China. Many fail to systematically incorporate patient perspectives or adequately address disease severity, potentially misaligning with patient needs and increasing long‐term healthcare costs [20, 21]. Although VAFs provide a theoretical basis for value, hospital formulary selection is an operational decision process constrained by local budgets and hospital capacity. More importantly, they are not designed to capture the unique institutional and policy contours of the Chinese healthcare system.

Within China, although general drug selection guidelines and preliminary VAF attributes have been proposed [22, 23], a critical gap remains: there is no standardized, operational, and consensus‐driven multi‐criteria decision framework specifically tailored for selecting innovative anticancer drugs in hospital formularies. The lack of a transparent, actionable tool that integrates the priorities of diverse domestic stakeholders—and that reflects realities such as the “tripartite reform” linking medical services, insurance, and pharmaceuticals—hampers consistent, evidence‐informed decision‐making across institutions.

To address this gap, this study aims to develop and prioritize a set of core decision criteria for the formulary selection of innovative anticancer drugs in China, rather than a purely normative value framework. Informed by established international VAFs and adapted to the local context through a structured, multi‐stakeholder Delphi consensus process, we seek to answer the following research questions:

1. What dimensions and indicators are considered essential by a multidisciplinary panel for evaluating the value of innovative anticancer drugs in Chinese hospital formularies?
2. What is the relative priority of these dimensions?
3. How can these criteria be structured into a practical decision‐making framework?

The resulting framework is intended to provide a transparent, structured, and justifiable tool to support formulary committees in reconciling trade‐offs among clinical benefit, patient‐centricity, economic impact, and system sustainability.

## 2 Methods

### 2.1 Study Design

This study was conducted in two phases: a comprehensive literature review to identify potential decision criteria, followed by a two‐round Delphi survey to establish consensus. The Delphi technique is a structured, iterative process designed to aggregate expert opinions on complex issues where definitive evidence is lacking [24, 25]. The study was approved by the Ethics Committee (Approval No. 2024‐KY‐2036‐002).

### 2.2 Literature Review

A comprehensive literature search was performed across PubMed, Embase, Cochrane Library, Web of Science, and CNKI (China National Knowledge Infrastructure) databases covering the period from 2000 to 2025. Search terms included “value,” “value assessment,” “value‐based,” “framework,” “drug selection,” “decision,” and “decision‐making.” Articles published in languages other than English or Chinese were excluded. The specific search strategy and screening criteria are provided in Supplemental Material A. Based on this review and the research team's practical experience, an initial set of 19 indicators spanning nine dimensions was compiled to form the draft framework for the first round of the Delphi survey.

### 2.3 Panel Selection and Participation

A multidisciplinary expert panel was purposively sampled to capture a broad spectrum of stakeholders and ensure the validity and applicability of the consensus. Participants were selected based on their professional expertise and regional representation to reflect the diversity of the Chinese healthcare landscape. The panel included:

- Clinicians: Heads of departments of oncology, breast surgery, and respiratory medicine from 11 tertiary medical institutions.
- Pharmacists: Heads of clinical pharmacy departments from the same institutions.
- Health Administrators and Researchers: Experts in health economics and health technology assessment (HTA) from seven domestic universities.
- Patient Representatives: One patient representative from each institution, selected based on their lived experience with cancer and understanding of anticancer drug therapy and disease management.

Geographical diversity was ensured by including experts from 10 provinces across the eastern, central, and western regions of China. In total, 85 experts were invited, and 62 provided valid responses in Round 1 (response rate: 72.9%).

### 2.4 Delphi Process

The Delphi survey was administered electronically using the “Questionnaire Star” platform, a widely used online survey tool in China. The study design and questionnaire were pretested by two external reviewers to ensure clarity and face validity. The internal consistency of the Round 1 questionnaire, as measured by Cronbach's α, was found to be 0.75, indicating acceptable reliability.

### 2.5 Round 1

The first‐round questionnaire presented an initial framework consisting of nine dimensions and 19 indicators. Participants were asked to rate the importance of each indicator on a five‐point Likert scale (1 = strongly disagree to 5 = strongly agree) and provide open‐ended comments along with suggestions for additional indicators. The questionnaire for round 1 is listed in Supporting Information S1: Table C.1.

### 2.6 Round 2

**Figure 1:**
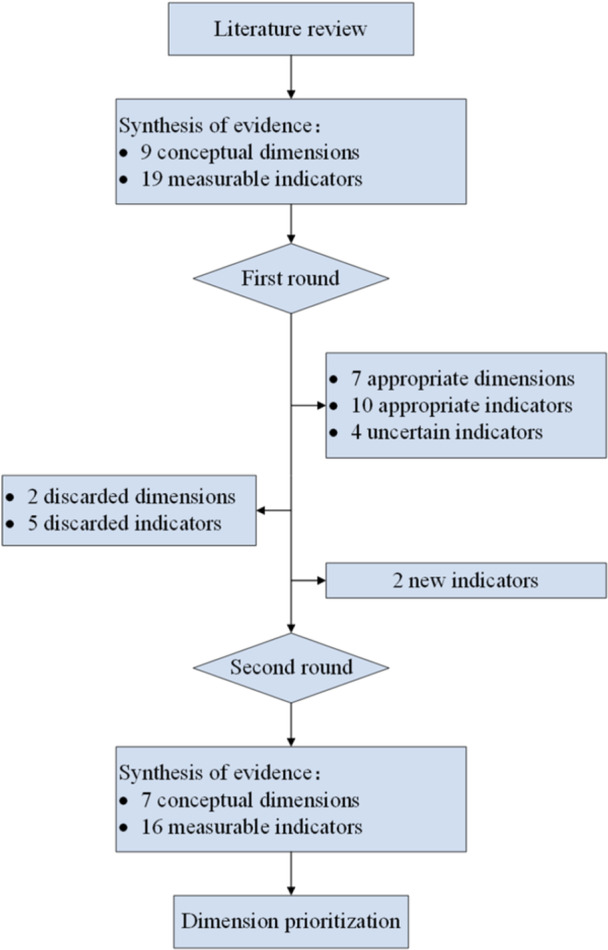
Flowchart of the Delphi process.

In the second round, a revised framework incorporating feedback from Round 1 was presented. Participants re‐evaluated both modified and newly proposed indicators while ranking the importance of the consolidated dimensions. The process concluded after two rounds due to responses demonstrating a high level of stability and consensus, with minimal further modifications suggested, indicating that response saturation had been achieved. The questionnaire for Round 2 is provided in Supporting Information S1: Table C.2. A flowchart of the Delphi process is presented in Figure 1.

Patient representatives participated in Round 1 to identify value‐relevant indicators but were not included in Round 2, which focused on technical tasks (e.g., refining operational definitions and measurement rules) requiring specialized clinical or HTA expertise. To assess potential bias, we compared first‐round ratings between patient and professional subgroups (Supporting Information S1: Table C.4).

### 2.7 Statistical Analysis

A Delphi round was considered valid if response rate ≥ 70%. Consensus was defined as ≥ 80% of respondents rating an indicator 4–5 for retention or 1–2 for exclusion, with remaining items revised for the next round; agreement dispersion was measured using the interquartile ranges (IQRs) [26], where an IQR ≤ 1 indicated strong agreement and ≤ 2 moderate agreement. [27]. Additionally, Kendall's coefficient of concordance (W) was calculated to evaluate the overall agreement and stability of expert opinions across the rounds. A W value exceeding 0.6 was interpreted as indicative of substantial agreement, and statistical significance was set at *p* < 0.05.

## 3 Results

### 3.1 Characteristics of Participants

A total of 62 invited experts completed the first‐round survey, yielding a response rate of 72.9%. In the second round, 51 participants submitted responses, reflecting an 82.3% retention rate relative to the first round (see Supplemental Material B). Patient representatives participated only in Round 1 per the study design. The demographic and professional characteristics of participants in both rounds are summarized in Table 1. The expert panel included diverse stakeholders such as physicians, pharmacists, health administrators, and researchers in health economics and HTA. Across both rounds, the majority of professional participants held positions at or above the intermediate level, exceeding 80% in each round. The demographic characteristics of the participants are presented in Table 1.

**Table 1:** Participant Characteristics.

Characteristic, *n* (%)	Round 1 *n* (%)	Round 2 *n* (%)
Specialty		
Respiratory	11(17.7%)	11(21.6%)
Breast	11(17.7%)	11(21.6%)
Oncology	11 (17.7%)	11 (21.6%)
Pharmacy	11 (17.7%)	11 (21.6%)
Pharmacoeconomics^a^	6 (9.7%)	6 (11.8%)
HTA^a^	7 (11.3%)	7 (13.7%)
N/A (Patient Representative)	11 (17.7%)	0 (0%)
Region of practice		
Beijing	1 (1.6%)	1 (2%)
Shanghai	2 (3.2%)	2 (4%)
Henan Province	15 (24.2%)	12 (23.5%)
Anhui Province	10 (16%)	8 (15.7%)
Sichuan Province	6 (9.7%)	5 (9.8%)
Jiangsu Province	6 (9.7%)	5 (9.8%)
Guangdong Province	6 (9.7%)	5 (9.8%)
Shandong Province	6 (9.7%)	5 (9.8%)
Heilongjiang Province	5 (8.1%)	4 (7.8%)
Yunnan Province	5 (8.1%)	4 (7.8%)
Age (years)		
18 ~ 30	8 (12.9%)	7 (13.7%)
31 ~ 40	19 (30.6%)	15 (29.4%)
41 ~ 50	25 (40.4%)	23 (45.1%)
51 ~ 60	8 (12.9%)	5 (9.8%)
61 ~ 70+	2 (3.2%)	1 (2%)
Gender		
Male	20 (32.3%)	19 (37.3%)
Female	42 (67.7%)	32 (62.7%)
Experience (years)		
0 ~ 5	19 (30.7%)	8 (15.7%)
5 ~ 10	11 (17.7%)	11 (21.6%)
10+	32 (51.6%)	32 (62.7%)

a Some participants hold multiple specialties.

### 3.2 First Round

The first Delphi round achieved consensus on 10 of the initial 19 indicators, with median scores ranging from 4 to 5. All indicators showed moderate to high levels of agreement, as evidenced by IQR of 2 or less. Detailed scoring results are presented in Supporting Information S1: Table C.1. Among the remaining nine indicators, four were designated for revision in the next round (median scores: 2.3–3.9), and five were considered unsuitable for inclusion (median scores ≤ 2.0). Qualitative feedback from participants consistently highlighted the importance of grounding indicators in objective, quantifiable, and publicly accessible data. This feedback, combined with formal proposals submitted by at least nine participants, informed the revision of the four indicators and the addition of two new indicators for further evaluation. Appendix Table C.4 presents a comparative assessment of the composite criterion across all indicators for patient representatives and the professional panel, revealing strong alignment on core dimensions, such as “Clinical Efficacy” and “Safety.” The Kendall's W for Round 1 was 0.62 (*p* < 0.001).

### 3.3 Second Round and Final Framework

The second‐round questionnaire introduced a revised framework comprising 7 dimensions and 16 indicators. The Kendall's for the second round improved to 0.86 (*p* < 0.001), demonstrating strong agreement. Full consensus was achieved on all 16 indicators. As shown in Table 2, median scores ranged from 4.0 to 5.0, with IQR values ranging from 0 to 1 across all indicators, indicating strong agreement. Key refinements include (1) revising the indicator “patient out‐of‐pocket expenditure” to “annual patient out‐of‐pocket expenditure” to enhance comparability across diverse healthcare contexts, and (2) standardizing the definitions and measurement protocols for the disease severity dimension—including tumor staging and pathological subtype—explicitly framing it as a population‐level prioritization criterion in formulary decision‐making, thereby enabling consistent, reliable, and clinically meaningful data extraction from electronic health records. A comparative summary of indicator changes across rounds is shown in Supporting Information S1: Table C.3.

**Table 2:** Final dimension ranking list with definition and agreement percentage.

Dimension	Definition	Indicator	Median (IQR)	Agreement%	Rank
Clinical benefits	The beneficial influence of pharmaceutical products on health.	Clinical efficacy	5.0 (1.0)	98.56	1
		Quality of life	5.0 (1.0)	98.57	
Safety	The relationship between benefit and risk.	Toxicological classification assessment	5.0 (1.0)	98.57	2
Disease severity	The level of malignancy and the extent of harm to the patient caused by the tumor.	Tumor staging	5.0 (1.0)	97.12	4
		Pathological type	5.0 (1.0)	95.68	
		Patient performance status	5.0 (1.0)	97.12	
Economic impact	The evaluation of the financial consequences of adopting the health technology on both the healthcare system (budget) and the patient (financial toxicity).	Patient out‐of‐pocket expenditure per year	4.0 (1.0)	92.09	3
		Treatment regimen costs per month	4.0 (1.0)	92.08	
		Cost‐effectiveness/utility analysis (CEA/CUA) results	5.0 (1.0)	94.25	
		Budget impact analysis results	4.0 (1.0)	92.80	
Disease rarity	The morbidity rate is less than 1/10,000, and the number of patients is less than 140,000.	Incidence of disease	4.0 (1.0)	83.45	7
Availability	The extent to which healthcare services and alternative therapies are accessible and obtainable for the target patient population.	Access barriers of alternative therapy	5.0 (1.0)	95.68	5
		Pharmaceutical access	5.0 (1.0)	94.97	
		Access barriers of genetic testing	4.0 (1.0)	94.24	
Quality and consistency of evidence	The quality and consistency of clinical trials and economic analysis evidence.	Quality of evidence	5.0 (1.0)	100	6
		Consistency of evidence	5.0 (1.0)	99.29	

**Figure 2:**
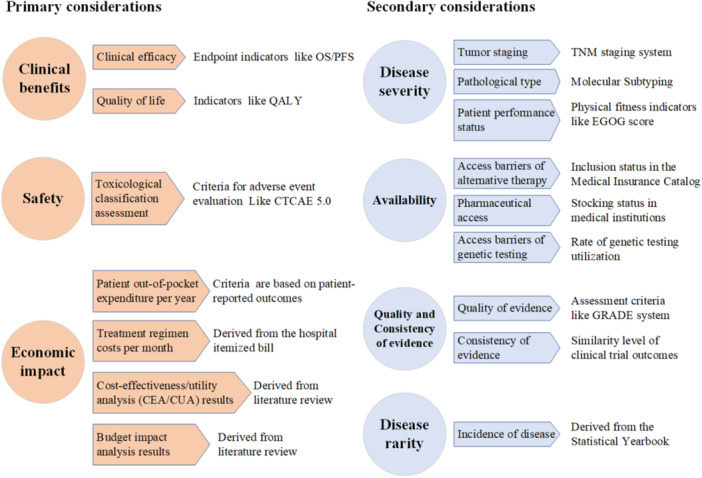
Decision‐making framework for formulary selection based on the Delphi consensus.

Additionally, participants established a clear hierarchy by ranking the relative importance of the seven decision‐making dimensions (see Table 2). To ensure transparent and standardized implementation, Table 3 specifies, for each indicator, its operational definition, primary data source, and the variable type. Clinical benefits, safety, and economic impact emerged as the top three prioritized dimensions. The final integrated framework, which aligns these ranked dimensions with their corresponding measurable indicators, is presented in Figure 2.

**Table 3:** Indicator operationalization table.

Indicator	Operational definition	Primary data source	Variable type
Clinical efficacy	Measuring “Does this treatment work?”	Clinical trial	Continuous/Ratio
Quality of life	The individual's subjective assessment of their overall living situation.	Literature	Continuous
Toxicological classification assessment	Grading of adverse reactions related to cancer treatment.	Drug Package Insert/Clinical Trial Publications	Ordinal
Tumor staging	Standardized assessment of tumor extent (T), lymph node involvement (N), and metastasis (M) to determine an overall stage.	EMR (Electronic Medical Record)	Ordinal
Pathological type	Categorization of tumors based on their microscopic appearance and established criteria.	EMR (Electronic Medical Record)	Categorical
Patient performance status	Standardized tool to assess functional status and overall health before cancer treatment.	Rating scale	Ordinal
Patient out‐of‐pocket expenditure	The annualized average cost borne by the patient after basic medical insurance reimbursement.	Patient report	Continuous
Treatment regimen costs per month	Total standardized cost of a treatment per month from a specified perspective	Hospital Itemized Bill	Continuous
Cost‐effectiveness/utility analysis (CEA/CUA) results	The quantified comparison of intervention costs to health outcomes gained.	Literature	Continuous
Budget impact analysis results	Estimated financial impact on a specific budget from adopting an intervention.	Literature	Continuous
Incidence of disease	Number of new cases during a specified time period.	Statistical Yearbook	Continuous
Access barriers of alternative therapy	Obstacles preventing patient access to alternative treatment options.	Medical Insurance Catalog	Binary
Pharmaceutical access	Conditions enabling patients to obtain and appropriately use needed medicines.	Institutional Formulary	Binary
Access barriers of genetic testing	Multilevel obstacles to obtaining recommended genetic testing.	HIS (Hospital Information System)	Binary
Quality of evidence	The degree of confidence that study findings reflect the true effect.	Literature	Ordinal
Consistency of evidence	Similarity level of clinical trial outcomes.	Literature	Ordinal

## 4 Discussion

To our knowledge, this Delphi study establishes the first multi‐stakeholder, consensus‐driven decision framework for formulary selection of innovative anticancer drugs within the context of the Chinese healthcare system. Through a structured process, we identified and prioritized 16 measurable indicators across seven key dimensions. Clinical benefits, safety, and economic impact were consistently ranked as the highest‐priority criteria by the expert panel. This prioritization closely reflects the core objectives of hospital formulary management, where the need to deliver effective and safe patient care must be balanced against constraints imposed by limited institutional budgets and the financial toxicity faced by patients [14, 23, 28, 29, 30]. While dimensions such as disease rarity and quality of evidence received lower rankings, their significance is not diminished; rather, they serve as critical differentiating factors when comparing therapies with similar performance in the primary criteria.

The refinement of the framework through the Delphi process highlights a pragmatic focus on actionability and measurability. The exclusion of PROs and equity indicators reflects a systemic gap in current data infrastructure rather than a lack of perceived importance. It highlights an urgent need for policymakers to mandate standardized collection of patient‐reported data to enable their inclusion in future value assessments [31, 32]. This outcome reflects the panel's deliberate prioritization of indicators that can be operationalized using objective, quantifiable data—such as tumor stage, treatment costs, and inclusion in insurance catalogs—thereby reducing subjectivity and enhancing the framework's applicability to real‐world decision‐making. However, this pragmatic trade‐off highlights a critical priority for health system improvement: strengthening the robustness and accessibility of patient‐centered data to match those of clinical data.

Our framework draws deliberate inspiration from established international VAFs, particularly the ISPOR Value Flower, while critically adapting them to the local context. Although the core elements of our framework—clinical benefit, safety, and cost‐effectiveness—correspond directly to the “core” petals of the Value Flower [14], its novelty stems from the context‐specific operationalization tailored to China's hospital formulary management system. Indicators such as “Budget Impact Analysis” (tied to hospital access) and “Access barriers of genetic testing” (assessing institutional capacity for companion diagnostics) directly shape whether an innovative drug can be feasibly adopted. This adaptation is not merely conceptual but is driven by China's ongoing “tripartite reform” of healthcare—a policy initiative that explicitly integrates reforms in medical services, medical insurance, and pharmaceutical supply [33]. Within this system, a drug's value depends not only on its clinical and economic attributes but also on its accessibility via national reimbursement mechanisms and its feasibility for implementation in hospital settings, which are often constrained by diagnostic capacity and budgetary control systems [33]. This systemic interplay represents a distinctive characteristic that many international frameworks, designed for different healthcare architectures, fail to explicitly account for.

Another significant strength of this study is the active engagement of a diverse multi‐stakeholder panel, including patient representatives from the foundational first round. This approach ensures that the resulting consensus integrates clinical expertise, administrative pragmatism, and patient perspectives, thereby addressing the limitations of previous studies that were often dominated by a single viewpoint [32, 34].

This framework provides hospital Pharmacy & Therapeutics (P&T) committees with a structured tool to navigate the complexities involved in selecting innovative anticancer agents. However, participants rightly emphasized the dynamic nature of these decisions. Factors such as the policy‐contingent nature of cost‐effectiveness evaluations [35] and the evolution of clinical benefit with longer‐term follow‐up [36, 37] require flexible application of the framework. Future applications should therefore incorporate systematic methodologies to account for inter‐indicator interactions and temporal shifts in evidence.

Furthermore, the framework integrates policy‐sensitive novel indicators, notably “Pharmaceutical Access.” Given the annual updates to China's National Reimbursement Drug List (NRDL), changes in medicine status are inevitable. To ensure decision‐making robustness and longitudinal consistency, participating institutions must explicitly document the NRDL version (and its effective date) applied during framework implementation—for instance, in new medicine selection—and align periodic framework reviews with national policy cycles (e.g., annually). This standardized temporal anchoring preserves cross‐cycle comparability and strengthens institutional readiness for scalable, policy‐responsive adoption across diverse healthcare settings.

While this study prioritized and ranked dimensions, it did not assign explicit weights. This framework provides the essential, consensus‐based set of criteria and their operational definitions required to construct a formal Multi‐Criteria Decision Analysis (MCDA) model and is therefore best viewed as a precursor and foundational input to MCDA. In the absence of formal weighting, hospital committees can adopt pragmatic approaches. For instance, they could apply the ranked dimensions as a tiered decision filter: first verifying that candidate drugs meet minimum acceptable thresholds on the “Primary Considerations,” and then using the “Secondary Considerations” to resolve ties. Alternatively, local stakeholders may conduct pragmatic weighting exercises—such as assigning tiered weights, allocating discrete points across dimensions, or performing pairwise comparisons via the Analytic Hierarchy Process (AHP)—grounded in this prioritized framework to derive context‐specific weights.

## 5 Limitations

This study has several principal limitations. First, although the expert panel was carefully selected for multidisciplinary expertise, its geographic representation focused predominantly on economically developed provinces (eastern and central China), where innovative anticancer drugs are predominantly adopted first in large tertiary hospitals in these regions due to greater economic resources and clinical capacity. This regional imbalance may limit the generalizability of the findings to healthcare settings in resource‐constrained regions. Future studies should validate and potentially recalibrate the framework in secondary hospitals and under‐resourced regions.

Second, the framework establishes priority dimensions but intentionally omits indicator‐specific weighting. The contextual variability of criteria importance across cancer types and treatment scenarios introduces interpretative challenges for direct drug comparisons. However, rigorous quantitative validation—grounded in MCDA principles—is still required to develop evidence‐based, transparent, and reproducible weighting algorithms.

Third, implementation requires validation through empirical studies. Although Delphi participants confirmed indicator measurability, the framework's operational effectiveness in hospital P&T committees—including its influence on formulary decisions and procedural efficiency—requires prospective evaluation using implementation science methods to identify adoption barriers and assess decision‐making transparency improvements. Furthermore, although patient representatives contributed to the initial development of the indicator pool in Round 1, their exclusion from the technical scoring in Round 2 is a limitation. However, the retention of patient‐prioritized indicators (e.g., quality of life, out‐of‐pocket expenditures) in the final consensus suggests that the multidisciplinary experts successfully aligned with patient values. Future iterations should explicitly integrate patient preferences into technical evaluation phases, such as scoring or weighting procedures.

## 6 Conclusions

This Delphi study establishes a systematic and pragmatic framework for formulary selection of innovative anticancer drugs in Chinese hospitals, particularly in resource‐adequate regions. By integrating multidisciplinary stakeholder consensus with internationally recognized value assessment principles, the framework provides a transparent and actionable tool to guide hospital decision‐making. It contributes to the academic field by offering a structured methodology for prioritizing drug value dimensions in resource‐constrained settings. For policy and practice, it delivers a standardized approach that supports the objectives of China's healthcare reform, promoting more equitable, efficient, patient‐centered and evidence‐informed resource allocation. Future research should focus on empirically validating and refining the framework through real‐world application and the development of context‐specific weighting systems.

## Author Contributions

**Jie Zhao:** methodology; project administration; writing – original draft; writing – review and editing. **Youhong Hu:** conceptualization; project administration; resources; writing – review and editing. **Bin Wu:** methodology; formal analysis. **Ming Hu:** methodology; formal analysis. **Yan Liang:** data curation; investigation; writing – review and editing. **Jia Li:** data curation; investigation; writing – review and editing. **Gaoxing Qiao:** data curation; investigation. **Xintong Fan:** data curation; investigation. **Helei Yan:** data curation; investigation. **Xiaoyun Wang:** data curation; investigation. **Zhixin Zheng:** data curation; investigation. **Xiaodan Zhang:** data curation; investigation. **Junwei Li:** investigation. **Tingting Lou:** investigation. **Yuchen Ma:** investigation.

## Ethics Statement

This study was approved by the First Affiliated Hospital of Zhengzhou University Ethics Committee (Approval No. 2024‐KY‐2036‐002).

## Conflicts of Interest

The authors declare no conflicts of interest.

## Supporting information


Supporting File

## Data Availability

The data that supports the findings of this study are available in the supporting material of this article.
