# Supplementary material for: A Delphi Study to Identify Priority Indicators for Formulary Selection of Innovative Anticancer Drugs in China
Source: Health Expect. 2026 Apr 11;29(2):e70652. doi: 10.1111/hex.70652 (PMC13069357; doi:10.1111/hex.70652)
Supplement: Supplementary file 1 — Supporting File [file HEX-29-e70652-s001.docx]

**Supplemental Materials**

**Supplemental** **Material A. Literature review process**

The search strategy combined two concept blocks: (1) value-related terms and (2) decision/framework terms for drug selection. Keywords were searched in titles/abstracts (and database keywords where available), using OR to combine synonyms within each block and AND to combine the blocks.

Eligibility Criteria

We included publications that described structured frameworks, criteria, or guidelines for evaluating drugs (general or oncology-specific) for formulary inclusion or value assessment. Publications were excluded if they: 1) did not list explicit decision criteria; 2) focused solely on economic evaluation without multi-criteria elements; 3) were not published in English or Chinese.

**PRISMA flow diagram**

**Identification of studies via databases and registers**

Records removed *before screening*:

Duplicate records removed (n = 366)

Records marked as ineligible by automation tools (n = 3032)

Records identified from:

Databases (n = 4892)

PubMed (n = 3600)

Cochrane Library (n = 827)

Web of Science (n = 453)

CNKI (n = 12)

**Identification**

Records excluded

(n = 1142)

Conferences or case reports (n = 804)

Not meeting study aim (n = 338)

Records screened

(n = 1494)

**Screening**

Reports sought for retrieval

(n = 352)

Reports not retrieved

(n = 88)

Reports excluded:

Reason 1 (n = 161)

Reason 2 (n = 25)

Reason 3 (n = 14)

Reports assessed for eligibility

(n = 264)

**Included**

Studies included in review

(n = 64)

Reports of included studies

(n = 64)

**Supplemental Material B. Delphi panel invitations and responses**

85 Invitation to participants Accepted 62

Delphi round 1 62 responded (72.94% response rate)

Delphi round 2 51 responded (82.26% response rate)

**Supplemental Material C. Supplemental Tables**

**Table C.1 Composition and results of the first round of the Delphi**

| **Dimension** | **Definition** | **Indicator** | **Measurement Method/Source** | **Literature Source** | **IQR** | **Median** |
| --- | --- | --- | --- | --- | --- | --- |
| Clinical benefit | The beneficial influence of pharmaceutical products on health. | Clinical efficacy | OS/PFS[long-term]; RR/ORR[short-term])* | Liu ITT et al.^1^  Zhang M et al.^2^  ISPOR Value Flower^3^ | 1 | 4 |
|  |  | Quality of life | QALY* |  | 1 | 5 |
|  |  | Patient-reported outcomes (PRO) | Patient's statement |  | 1 | 2 |
| Safety | The relationship between benefit and risk. | Toxicological classification assessment | CTCAE 5.0* | ASCO framework^4^  ESMO-MCBS^5^  NCCN framework^6^ | 1 | 5 |
|  |  | Incidence rate of adverse drug reactions (ADR) | ADR report |  | 1 | 2 |
|  |  | Adverse effects in the short-term and long-term | Clinical observation report |  | 1 | 2 |
| Disease severity | The level of malignancy and the extent of harm to the patient caused by the tumor. | Tumor staging | Stage I - IV | Zhang M et al.^2^  Li ZX et al. ^7^  ISPOR Value Flower^3^ | 1 | 5 |
|  |  | Transfer site | Site of metastasis |  | 1 | 2.4 |
|  |  | Mortality rate | Statistical Yearbook |  | 2 | 2.5 |
| Economic impact | The evaluation of the financial consequences of adopting the health technology on both the healthcare system (budget) and the patient (financial toxicity). | Out-of-pocket costs to patients | Patient report | ASCO framework^4^  Zhang M et al.^2^ | 2 | 3.8 |
|  |  | Treatment regimen costs per month | Hospital Itemized Bill |  | 1 | 5 |
|  |  | Cost-effectiveness/utility analysis (CEA/CUA) results | Literature review |  | 1 | 5 |
|  |  | Affordability | Patient report |  | 2 | 2.8 |
| Disease rarity | The morbidity rate is less than 1/10,000, and the number of patients is less than 140,000. | Incidence of disease | Statistical Yearbook | Zhang M et al.^2^  Guo J et al.^8^ | 1 | 5 |
| Equity | The impacts and trade-offs of this health technology for all patient groups. | The health technology can be accessed by all patient groups | —— | ISPOR Value Flower^3^  Zhang M et al.^2^  Gauvreau CL et al.^9^ | 1 | 2 |
| Availability | The extent to which healthcare services and alternative therapies are accessible and obtainable for the target patient population. | Access barriers of alternative therapy | Inclusion status in the Medical Insurance Catalog | Zhang M et al.^2^  Smith S et al.^10^ | 1 | 4 |
| Quality and consistency of evidence | The quality and consistency of clinical trials and economic analysis evidence. | Quality of evidence | GRADE system* | NCCN framework^6^  Zhang M et al.^2^ | 1 | 4 |
|  |  | Consistency of evidence | Literature review |  | 1 | 4 |
| Risk preference | Differences in patients’ risk tolerance. | The risk acceptance level of the patient's willingness to adopt the health technology | DCE/BWS* | ISPOR Value Flower^3^ | 1 | 2 |

* OS: Overall Survival, length of time from either the date of diagnosis or the start of treatment for a cancer，that patients diagnosed with the disease are still alive; PFS: Progression-Free Survival, length of time during and after the treatment of a cancer，that a patient lives with the disease but it does not get worse; RR: Relative Risk, the ratio of the risk of an event occurring in an exposed group versus an unexposed group; ORR: Objective Response Rate, percentage of people in a treatment group who have a partial or complete response to the treatment within a certain period of time. QALY: Quality-Adjusted Life Year. CTCAE: Common Terminology Criteria for Adverse Events version 5.0. DCE: Discrete Choice Experiment. BWS: Best–Worst Scaling.

**Table C.2 Results of the second round of the Delphi**

| Dimension | Definition | Indicator | Measurement Method/Source | IQR | Median |
| --- | --- | --- | --- | --- | --- |
| Clinical benefit | The beneficial influence of pharmaceutical products on health. | Clinical efficacy | OS/PFS[long-term]; RR/ORR[short-term]) | 1 | 5 |
|  |  | Quality of life | QALY | 1 | 5 |
| Safety | The relationship between benefit and risk. | Toxicological classification assessment | CTCAE 5.0 | 1 | 5 |
| Disease severity | The level of malignancy and the extent of harm to the patient caused by the tumor. | Tumor staging | Stage I - IV | 1 | 5 |
|  |  | Pathological type | Molecular Subtyping | 1 | 5 |
|  |  | Patient performance status | ECOG score | 1 | 5 |
| Economic impact | The evaluation of the financial consequences of adopting the health technology on both the healthcare system (budget) and the patient (financial toxicity). | Patient out-of-pocket expenditure per year | Patient report | 1 | 4 |
|  |  | Treatment regimen costs per month | Hospital Itemized Bill | 1 | 4 |
|  |  | Cost-effectiveness/utility analysis (CEA/CUA) results | Literature review | 1 | 5 |
|  |  | Budget impact analysis results | Literature review | 1 | 4 |
| Disease rarity | The morbidity rate is less than 1/10,000, and the number of patients is less than 140,000. | Incidence of disease | Statistical Yearbook | 1 | 4 |
| Availability | The extent to which healthcare services and alternative therapies are accessible and obtainable for the target patient population. | Access barriers of alternative therapy | Inclusion status in the Medical Insurance Catalog | 1 | 5 |
|  |  | Pharmaceutical access | Inclusion status in the Institutional Formulary | 1 | 5 |
|  |  | Access barriers of genetic testing | Rate of genetic testing utilization | 1 | 4 |
| Quality and consistency of evidence | The quality and consistency of clinical trials and economic analysis evidence. | Quality of evidence | GRADE system | 1 | 5 |
|  |  | Consistency of evidence | Literature review | 1 | 5 |

**Table C.3** **Comparison between the proposed and final list of dimensions and criteria**

| Proposed List (Round 1) | | | Proposed List (after Round 2) | | |
| --- | --- | --- | --- | --- | --- |
| Dimension | **Indicator** | **Measurement method/Source** | **Dimension** | **Indicator** | **Measurement method/Source** |
| Clinical benefit | Clinical efficacy | OS/PFS[long-term]; RR/ORR[short-term]) | Clinical benefit | Clinical efficacy | OS/PFS[long-term]; RR/ORR[short-term]) |
|  | Quality of life | QALY |  |  |  |
|  | Patient-reported outcomes (PRO) | Patient's statement |  | Quality of life | QALY |
| Safety | Toxicological classification assessment | CTCAE 5.0 | Safety | Toxicological classification assessment | CTCAE 5.0 |
|  | Incidence rate of adverse drug reactions (ADR) | ADR report |  |  |  |
|  | Adverse effects in the short-term and long-term | Clinical observation report |  |  |  |
| Disease severity | Tumor staging | Stage I - IV | Disease severity | Tumor staging | TNM staging |
|  | Transfer site | Site of metastasis |  | Pathological type | Molecular Subtyping |
|  | Mortality rate | Statistical Yearbook |  | Patient performance status | ECOG score |
| Economic impact | Out-of-pocket costs to patients | Patient report | Economic impact | Patient out-of-pocket expenditure per year | Patient report |
|  | Treatment regimen costs per month | Hospital Itemized Bill |  | Treatment regimen costs per month | Hospital Itemized Bill |
|  | Cost-effectiveness/utility analysis (CEA/CUA) results | Literature review |  | Cost-effectiveness/utility analysis (CEA/CUA) results | Literature review |
|  | Affordability | Patient report |  | Budget impact analysis results | Literature review |
| Disease rarity | Incidence of disease | Statistical Yearbook | Disease rarity | Incidence of disease | Statistical Yearbook |
| Availability | Access barriers of alternative therapy | Inclusion status in the Medical Insurance Catalog | Availability | Access barriers of alternative therapy | Inclusion status in the Medical Insurance Catalog |
|  |  |  |  | Pharmaceutical access | Inclusion status in the Institutional Formulary |
|  |  |  |  | Access barriers of genetic testing | Rate of genetic testing utilization |
| Quality and consistency of evidence | Quality of evidence | GRADE system | Quality and consistency of evidence | Quality of evidence | GRADE system |
|  | Consistency of evidence | Similarity level of clinical trial outcomes |  | Consistency of evidence | Literature review |
| Equity | The health technology can be accessed by all patient groups | —— |  | | |
| Risk preference | The risk acceptance level of the patient's willingness to adopt the health technology | DCE/BWS |  | | |

**Table C.4 Median scores and IQRs for Round-1 indicator ratings by patient and professional participants**

| **Dimension** | **Indicator** | **Median** | | **IQR** | |
| --- | --- | --- | --- | --- | --- |
|  |  | **Patient** | **Professionals** | **Patient** | **Professionals** |
| Clinical benefit | Clinical efficacy | 4.3 | 4.7 | 1 | 1 |
|  | Quality of life | 5 | 4.8 | 1 | 1 |
|  | Patient-reported outcomes (PRO) | 2 | 2 | 1 | 1 |
| Safety | Toxicological classification assessment | 5 | 5 | 2 | 1 |
|  | Incidence rate of adverse drug reactions (ADR) | 2 | 2 | 2 | 1 |
|  | Adverse effects in the short-term and long-term | 2 | 2 | 1 | 1 |
| Disease severity | Tumor staging | 5 | 5 | 1 | 1 |
|  | Transfer site | 2.8 | 2.4 | 2 | 1 |
|  | Mortality rate | 3 | 2.5 | 2 | 1 |
| Economic impact | Out-of-pocket costs to patients | 4 | 3.8 | 2 | 1 |
|  | Treatment regimen costs per month | 5 | 5 | 1 | 1 |
|  | Cost-effectiveness/utility analysis (CEA/CUA) results | 5 | 5 | 2 | 1 |
|  | Affordability | 2 | 2.8 | 2 | 1 |
| Disease rarity | Incidence of disease | 5 | 5 | 1 | 1 |
| Availability | Access barriers of alternative therapy | 5 | 4 | 2 | 1 |
| Quality and consistency of evidence | Quality of evidence | 4 | 4 | 2 | 1 |
|  | Consistency of evidence | 4 | 4 | 2 | 1 |
| Equity | The health technology can be accessed by all patient groups | 2 | 2 | 2 | 1 |
| Risk preference | The risk acceptance level of the patient's willingness to adopt the health technology | 4 | 2 | 2 | 1 |

References

1. Liu ITT, Kesselheim AS, Cliff ERS. Clinical Benefit and Regulatory Outcomes of Cancer Drugs Receiving Accelerated Approval. *JAMA*. 2024;331(17):1471-1479. doi:10.1001/jama.2024.2396

2. Zhang M, Bao Y, Yang Y, Kimber M, Levine M, Xie F. Identifying Attributes for a Value Assessment Framework in China: A Qualitative Study. *PharmacoEconomics*. 2023;41(4):439-455. doi:10.1007/s40273-022-01235-6

3. Neumann PJ, Garrison LP, Willke RJ. The History and Future of the “ISPOR Value Flower”: Addressing Limitations of Conventional Cost-Effectiveness Analysis. *Value Health*. 2022;25(4):558-565. doi:10.1016/j.jval.2022.01.010

4. Schnipper LE, Davidson NE, Wollins DS, et al. Updating the American Society of Clinical Oncology Value Framework: Revisions and Reflections in Response to Comments Received. *J Clin Oncol*. 2016;34(24):2925-2934. doi:10.1200/JCO.2016.68.2518

5. Cherny NI, Dafni U, Bogaerts J, et al. ESMO-Magnitude of Clinical Benefit Scale version 1.1. *Ann Oncol*. 2017;28(10):2340-2366. doi:10.1093/annonc/mdx310

6. Carlson RW, Jonasch E. NCCN Evidence Blocks. *J Natl Compr Cancer Netw JNCCN*. 2016;14(5 Suppl):616-619.

7. Li ZX, Zhang Y, Zhang LY, Duan R. 2022 Semimonthly Table of Contents Drug selection guideline for medical institutions. CHINA PHARMACY. 2022;(07 vo 33):769-776.

8. Guo J, Liu P, Chen L, et al. National Rare Diseases Registry System (NRDRS): China’s first nation-wide rare diseases demographic analyses. *Orphanet J Rare Dis*. 2021;16(1):515. doi:10.1186/s13023-021-02130-7

9. Gauvreau CL, Schreyer L, Gibson PJ, et al. Development of a Value Assessment Framework for Pediatric Health Technologies Using Multicriteria Decision Analysis: Expanding the Value Lens for Funding Decision Making. *Value Health J Int Soc Pharmacoeconomics Outcomes Res*. Published online March 2024. doi:10.1016/j.jval.2024.03.012

10. Smith S, Connolly S. Re-thinking unmet need for health care: introducing a dynamic perspective. *Health Econ Policy Law*. 2020;15(4):440-457. doi:10.1017/S1744133119000161
